# Why genetic validation is crucial for identifying larval stages of *Arctica islandica* (Venerida, Arcticidae)

**DOI:** 10.3897/zookeys.1291.195659

**Published:** 2026-09-02

**Authors:** Chiraz A. Chaibi, Katharina Kniesz, Michael L. Zettler

**Affiliations:** 1 University of Rostock, Rostock, Germany University of Rostock Rostock Germany; 2 Leibniz Institute for Baltic Sea Research Warnemünde (IOW), Rostock, Germany Leibniz Institute for Baltic Sea Research Warnemünde (IOW) Rostock Germany

**Keywords:** Baltic Sea, Bivalvia, COI barcoding, larval development, morphology, ocean quahog, scanning electron microscopy

## Abstract

The ocean quahog *Arctica islandica* is a long-lived marine bivalve widely distributed in the western Baltic Sea and commonly used as a model organism in ecological and climatic research. Despite its importance, aspects of its early development remain poorly understood. This study attempts to provide a morphological description of *A. islandica* larvae. Plankton samples were collected in Kiel Bay (Baltic Sea) in June 2024, September 2024, and April 2025, using a WP2 net (mesh size 100 µm). Larvae were identified by sequencing the mitochondrial cytochrome oxidase subunit I (COI) gene and subsequently documented using light and scanning electron microscopy (SEM). A benthic juvenile was additionally examined for comparison. Larvae of *A. islandica* were detected exclusively in June and September, yielding a total of 17 COI sequences. Genetically confirmed veliger larvae were detected in September (shell length 143–185 µm), whereas larger larvae interpreted as pediveligers were detected in a separate sampling event in June (shell length 217–505 µm). Larval anatomical structures and the apparent absence of certain organs are discussed. This study provides the first genetically confirmed description of the veliger and pediveliger stages of *A. islandica* and demonstrates that morphology alone is insufficient for reliable identification of *A. islandica* larvae and emphasizes the necessity of genetic validation.

## Introduction

*Arctica islandica* (Linnaeus, 1767) is the only extant member of the family Arcticidae R.B. Newton, 1891 (1844) (Nicol 1951; Abbott 1974). Widely distributed in the coastal waters of the North Atlantic (Rowell et al. 1990), including the North Sea (Witbaard and Bergman 2003) and the Baltic Sea (Zettler and Alf 2021), it inhabits muddy to sandy–muddy sediments at depths from shallow coastal waters to 500 m (Nicol 1951; Rowell et al. 1990). The species tolerates temperatures between 0 °C and 19 °C and is considered boreal due to its sensitivity to subzero temperatures (Nicol 1951).

**Figure 1.**
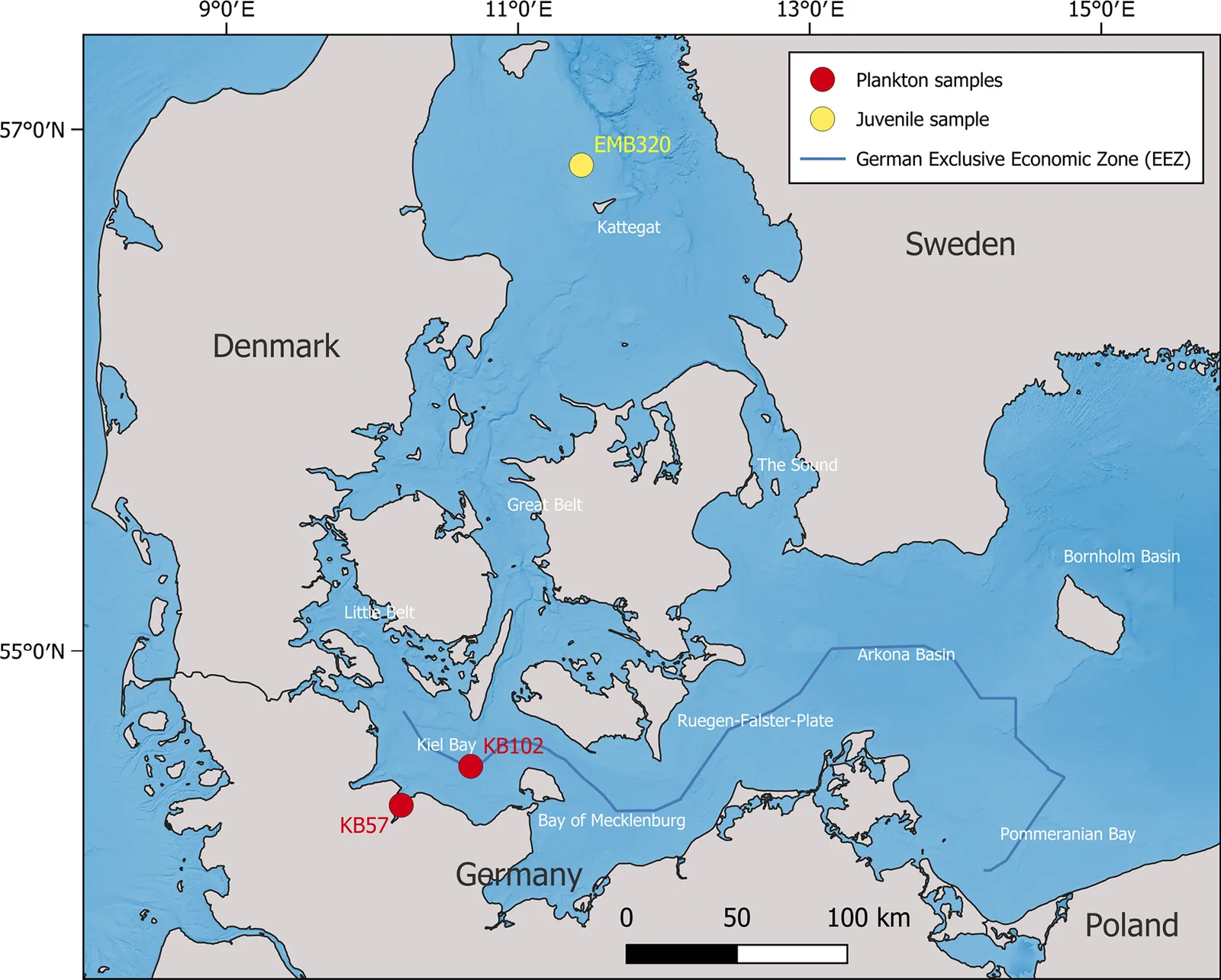
Map showing the three sampled plankton stations (by WP2 net) and the one benthic sample providing the juvenile specimens (by van Veen grab). Basemap: EMODnet Bathymetry World Base Layer, version 1, EMODnet Bathymetry Consortium (GGS Geo Consultancy 2024).

In the Baltic Sea, individuals are generally smaller and shorter-lived than in other parts of the species’ distribution range (Zettler et al. 2001; Zettler and Alf 2021). As a semi-enclosed inland sea, the Baltic Sea exposes marine organisms to elevated environmental stress, with climate-change effects already pronounced and expected to intensify (Reusch et al. 2018). The distribution of *A. islandica* in the Baltic Sea is constrained primarily by reduced salinity and oxygen availability (Mohrholz et al. 2015; Schulz et al. 2025). The species occurs across a salinity range of approximately 7–35 PSU, with its eastern distribution limit in the Arkona Basin (Fig. 1) reflecting the pronounced salinity gradient of the Baltic Sea (Darr et al. 2014; Zettler and Alf 2021). Deep anoxic basins such as the Gotland Basin are avoided (Conley et al. 2002). While adult individuals show considerable tolerance to hypoxic conditions (Theede et al. 1969; Theede 1973), larval stages are assumed to be more sensitive (Zettler et al. 2001).

*Arctica islandica* is renowned for its exceptional longevity, with maximum ages exceeding 400 years (Ridgway and Richardson 2011), whereas the oldest accounted individual is residing at 507 years (Butler et al. 2013). Growth is rapid during early life and slows markedly after sexual maturity (Thompson et al. 1980a), which individuals reach relatively late in their lifespan, attaining reproductive age after more than a decade (Thompson et al. 1980b; Zettler and Alf 2021). The species is dioecious, and the timing of gonadal development and spawning varies considerably among individuals (Thompson et al. 1980a), likely reflecting differences in growth and environmental conditions (Thompson et al. 1980b). Oocytes and spermatocytes are released into the water column primarily between May and August (von Oertzen 1972), and fertilization occurs externally. Multiple reproductive and developmental stages may coexist within a population, both spatially and temporarily (Loosanoff 1953; Thorarinsdóttir 2000).

Following fertilisation, bivalve larvae develop pelagically before undergoing metamorphosis to a benthic juvenile stage. Despite occurring at high densities in the water column, larval mortality is high (Hines 1986). The larval development rates are strongly temperature dependent. For *A. islandica*, development until metamorphosis requires several weeks, with shorter durations at higher temperatures (Landers 1972, 1976; Lutz et al. 1981, 1982). Larvae have been reported in the water column primarily between late spring and autumn, with peak abundances varying regionally (Mann 1985, 1989).

Larval development comprises three main stages: trochophore, veliger, and pediveliger. The trochophore stage is very short (between hours and a few days) and ends with the formation of prodissoconch I and velum for motility in the next phase. The veliger stage is characterised by the formation of the prodissoconch II. The pediveliger stage is characterised by the development of a foot and becomes competent for settlement and metamorphosis (Loosanoff et al. 1966; Gosling 2007). Metamorphosis involves extensive morphological reorganisation (Gosling 2007) and marks the transition to benthic life.

The identification of planktonic bivalve larvae based solely on morphology is often ambiguous due to strong similarities among taxa (Loosanoff et al. 1966; Chanley and Andrews 1971; Hendriks et al. 2005; Lutz et al. 2018). Consequently, molecular methods such as DNA barcoding have become essential for reliable species identification.

The larval stages of *A. islandica* have so far been insufficiently described and lack genetic confirmation, particularly in the Baltic Sea. The aims of this study are therefore (1) to provide a detailed morphological description of veliger and pediveliger larvae of *A. islandica*, (2) to genetically confirm species identity using mitochondrial COI sequences, and (3) to document the occurrence of larval stages in the western Baltic Sea. This work provides a basis for future studies on early life history and larval ecology of *A. islandica*.

## Methods

### Sampling and fixation

All plankton samples examined in this study were collected in Kiel Bay (Baltic Sea) during three different months of the year (Table 1; Fig. 1). Sampling was conducted using a WP2 closing net with a mesh size of 100 µm. The net was hauled vertically through the entire water column, thereby collecting organisms from all depths at each station.

**Table 1.** Sampled plankton stations using a WP2 net, including date of sampling, name of expedition, research vessel, station number, coordinates, water depth, salinity and water temperature.

**Date**	**Expedition**	**Research vessel**	**Station**	**Latitude, longitude**	**Depth (m)**	**Salinity (g/l)**	**Temperature (°C)**
**22.06.2024**	EMB343/ Legra24	Elisabeth Mann Borgese	KB57	54°23.22'N, 10°11.86'E	21.7	24.4	10.4
**03.09.2024**	ArKoBi24	Fortuna Kingfisher	KB102	54°32.52'N, 10°40.47'E	20.9	23.2	13.2
**13.04.2025**	EMB363/ ArKoBi25	Elisabeth Mann Borgese	KB102	54°32.58'N, 10°40.63'E	20.4	16.9	5.6

Samples collected in June and September were fixed in 96% ethanol and stored at −20 °C, whereas the April sample was examined immediately for live bivalve larvae. As no fixation was required for this examination, the sample was not preserved. During each sampling event, salinity and water temperature were recorded near the seafloor using a CTD probe (Table 1).

In addition to the plankton samples, a sediment sample collected on 1 July 2023 in the Kattegat (Baltic Sea) using a van Veen grab sampler aboard the RV Elisabeth Mann Borgese (station number EMB320_49_Mo2; 56°51.97'N, 11°26.01'E; temperature: 11.7 °C; salinity: 30.3 g/l; depth: 13.4 m) was used from the project “MGF Ostsee”. The sediment had been rinsed through a sieve (mesh size: 1 mm) and fixed with a 4% formaldehyde solution. Juvenile benthic individuals of *A. islandica* originating from this sample were included in the present study solely as reference material for shell development because juvenile *A. islandica* are rarely encountered in benthic samples.

An overview of all sampled larvae is provided in the supplementary material (Suppl. material 1: table SS1).

### Molecular analyses

To confirm that the examined specimens were larvae of *A. islandica*, molecular identification was carried out using DNA barcoding. A total of 85 larvae were transferred directly into PCR tubes. To facilitate DNA release, 51 larvae were carefully pierced prior to DNA extraction. In addition, 34 larvae were processed intact to preserve their morphology for subsequent illustration and larval stage description and to allow direct comparison between morphological and molecular identification.

Following complete evaporation of ethanol, DNA was extracted using 30 µl of Chelex® (Bio-Rad InstaGene™ Matrix) according to the manufacturer’s protocol. The mitochondrial cytochrome c oxidase subunit I (COI) gene was amplified by polymerase chain reaction (PCR).

Each PCR reaction contained 6.25 µl AccuStart™ ToughMix, 0.5 µl of each species-specific primer (AIS-COIF and AIS-COIR; García-Souto and Pasantes 2018), 4 µl nuclease-free water, and 0.25 µl GelTrack loading dye. The PCR cycling conditions consisted of an initial denaturation at 94 °C for 3 min, followed by 40 cycles of 94 °C for 30 s, 55 °C for 45 s, and 72 °C for 45 s, with a final extension at 72 °C for 5 min.

To enable standardized sequencing, the primers were equipped with the tails M13F-pUC (-40): GTTTTCCCAGTCACGAC and M13R-pUC (-40): CAGGAAACAGCTATGAC. PCR products were purified using ExoSAP-IT™ (Applied Biosystems) and sent to Macrogen Europe B.V. (Amsterdam, Netherlands) for bidirectional Sanger sequencing.

Sequence processing was carried out using Geneious Prime® 2026.0.1. Individual sequences were trimmed and assembled, resulting in 17 sequences, which were aligned using MAFFT v. 7.490 (Katoh and Standley 2013). Species identity was verified by comparison with reference sequences using the Basic Local Alignment Search Tool (BLAST; Altschul et al. 1990) provided by the National Center for Biotechnology Information (NCBI).

All sequences and associated metadata (sampling information, developmental stage, photographs, and raw read data) were deposited in the Barcode of Life Datasystems (BOLD; Ratnasingham et al. 2024) under the dataset “DS-ARKO01” (https://doi.org/10.5883/DS-ARKO01) and uploaded to GenBank (accession numbers PZ721695–PZ721711).

### Photography, measurement, and illustration

Specimens were examined using a Zeiss Axio Lab.A1 microscope and photographed with an AxioCam ERc 5c camera. Image processing was performed using ZEN 2.3 Lite software. Shell length and height of 47 larvae were measured from anterior to posterior margin following Gaspar et al. (2002) (Suppl. material 1: table SS1). For 14 larval specimens, the length and height of prodissoconch I and II, as well as hinge length, in addition to prodissoconch I and II of three juvenile specimens were measured (Suppl. material 1: table S2). Measurements were taken from photographs (prodissoconch I, II and hinge length from SEM images) using the ruler tool in Adobe® Photoshop® v. 26.8.1. The resulting measurements were visualised by a box plot generated in R v. 4.5.0 (R Core Team 2025) using the package “graphics”.

A representative larva of *A. islandica* was illustrated based on the captured images. The corresponding photograph was imported into Adobe Illustrator 2025 for iOS, where the contours of the specimen were digitally traced. Multiple layers with varying opacity were used to depict structural features. During the illustration process, the specimen was repeatedly re-examined under the microscope to ensure accurate representation of anatomical details. Internal anatomical interpretation is based on optical contrast and comparison with published larval descriptions. The larva is shown in dorsal view through the shell.

### Scanning electron and confocal laser scanning microscopy

Surface structures of the larvae were examined using scanning electron microscopy (SEM, Suppl. material 1: table SS1). Fourteen well-preserved specimens were selected, mounted on adhesive SEM stubs, and then sputter-coated with gold. Imaging was performed using a Zeiss Gemini MERLIN VP Compact SEM.

In addition, one representative specimen was selected for confocal laser scanning microscopy (CLSM, Suppl. material 1: table SS1). The specimen was slowly treated with acid to make it more translucent and then stained overnight with a 1:1 solution of acid fuchsin and Congo red. It was subsequently temporarily mounted on a microscope slide using glycerine. The animal was photographed from both the right and left sides.

CLSM imaging was performed using a Leica TCS SP5 system equipped with a Leica DM5000 B upright microscope and visible-light lasers (DPSS 10 mW 561 nm; HeNe 10 mW 633 nm; Ar 100 mW 458 nm, 476 nm, 488 nm, and 514 nm), controlled with LAS AF 2.2.1 software at the German Center for Marine Biodiversity (DZMB), Senckenberg am Meer, Wilhelmshaven following the procedure of Kihara and Rocha (2013). The stacked CLSM images are provided in the supplements (Suppl. material 1: fig. S1).

## Results

### Evidence of *Arctica islandica* larvae in Kiel Bay

**Figure 2.**
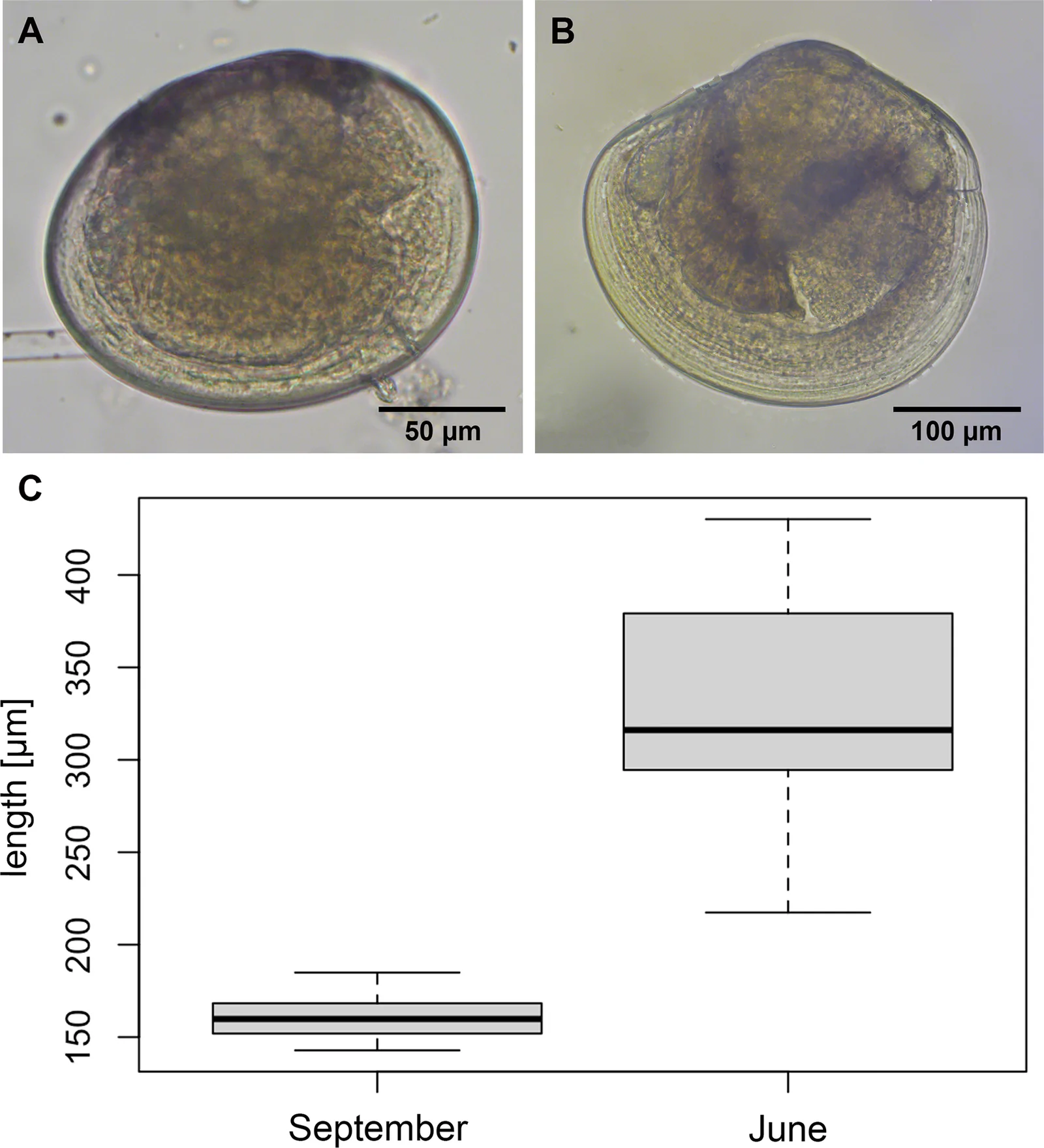
Detected larvae of *A. islandica* in Kiel Bay. **A, C**. In September; **B, C**. In June; **A**. Veliger larva (specimen Arko_L088), top view of left valve; **B**. Pediveliger larva (specimen Arko_L073), top view of left valve; **C**. Box plot of shell lengths of larvae collected during two independent sampling events: September (*n* = 10) and June (*n* = 37).

Larval abundance in the Kiel Bay varied between sampling events. The sample from June contained a high density of bivalve larvae that could be morphologically assigned to *A. islandica* (Fig. 2B). In contrast, the sample from September contained only a few bivalve larvae, morphologically consistent with *A. islandica* but with a more elongated shell (Fig. 2A) compared to the June specimens. No bivalve larvae were detected in the April 2025 sample during examination of the live material.

A total of 17 high-quality mitochondrial COI sequences (maximum length: 584 base pairs) were obtained from the larvae, confirming their identity as *A. islandica*. PCR success rates varied among DNA extractions. These included nine sequences from pierced larvae (June sample), seven sequences from intact larvae (June sample), and one sequence from an intact larva sampled in September. Successful identifications were therefore obtained from both pierced and intact specimens. These sequences were derived from specimens collected during both sampling periods (Suppl. material 1: table S3).

### Determination of the larval stage

The larvae collected in September ranged from 143 to 185 µm in length (Fig. 2C; *n* = 10; mean length of 162 µm; median 161 µm; interquartile range 17 µm) (Suppl. material 1: table S2). Lengths of larvae collected in June ranged from 217 µm to 505 µm (Fig. 2C; *n* = 37; mean length of 338 µm; median 327 µm; interquartile range 85 µm), indicating most individuals were at a similar developmental stage (Suppl. material 1: table SS1). The data are summarised in a boxplot (Fig. 2C).

### Description of *Arctica islandica* larvae

Systematics:

Class Bivalvia Linnaeus, 1758

Subclass Autobranchia Grobben, 1894

Superfamily Arcticoidea R.B. Newton, 1891 (1844)

Family Arcticidae R.B. Newton, 1891 (1844)

Genus *Arctica* Schumacher, 1817

### *Arctica islandica* Linnaeus, 1767

#### Veliger stage (Figs 2A, 2C, 3, 4)

**Material examined**. • 12 individuals, Baltic Sea, Kiel Bay, 54°32.52000'N, 10°40.47000'E, depth 20.9 m, 3 September 2024, Station KB102; specimens see Suppl. material 1: table SS1.

**Description. *Shell***. Prodissoconch I observed length 102–115 µm, height 78–91 µm (Fig. 4, Suppl. material 1: table S2), equivalve, equilateral; outline ovate, with straight, truncated dorsal margin; sculpture of irregular shallow pits and commarginal rims towards margin; fine radiating lines at margin of prodissoconch II (Fig. 4). Prodissoconch II observed length 143–185 µm (Fig. 2C; mean 162 µm, *n* = 10), height 110–141 µm, equivalve, subequilateral, narrow, elliptical, thin and translucent, slightly yellow; outer ventral area translucent to mantle edge; sculpture with irregular commarginal furrows; slight differences in convexity on either side of umbo; teeth and external ligament absent.

**Figure 3.**
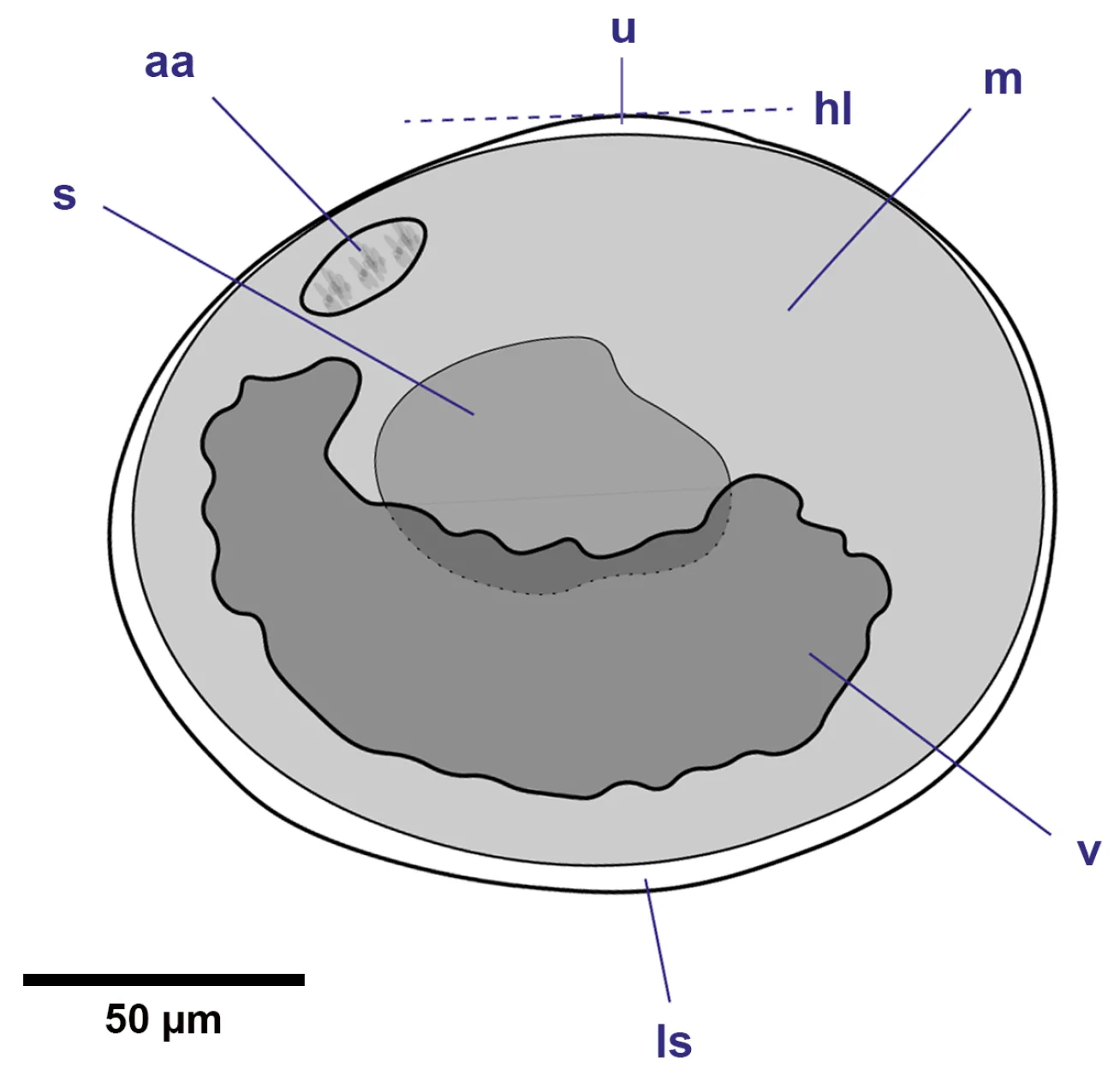
Illustration of veliger larva of *A. islandica* (specimen Arko_L088), view from left side of the shell; right: anterior, left: posterior, top: dorsal, bottom: ventral. Abbreviations: s = stomach, ls = larval shell, v = velum, m = mantle, hl = hinge line, u = umbo, aa = anterior adductor.

**Figure 4.**
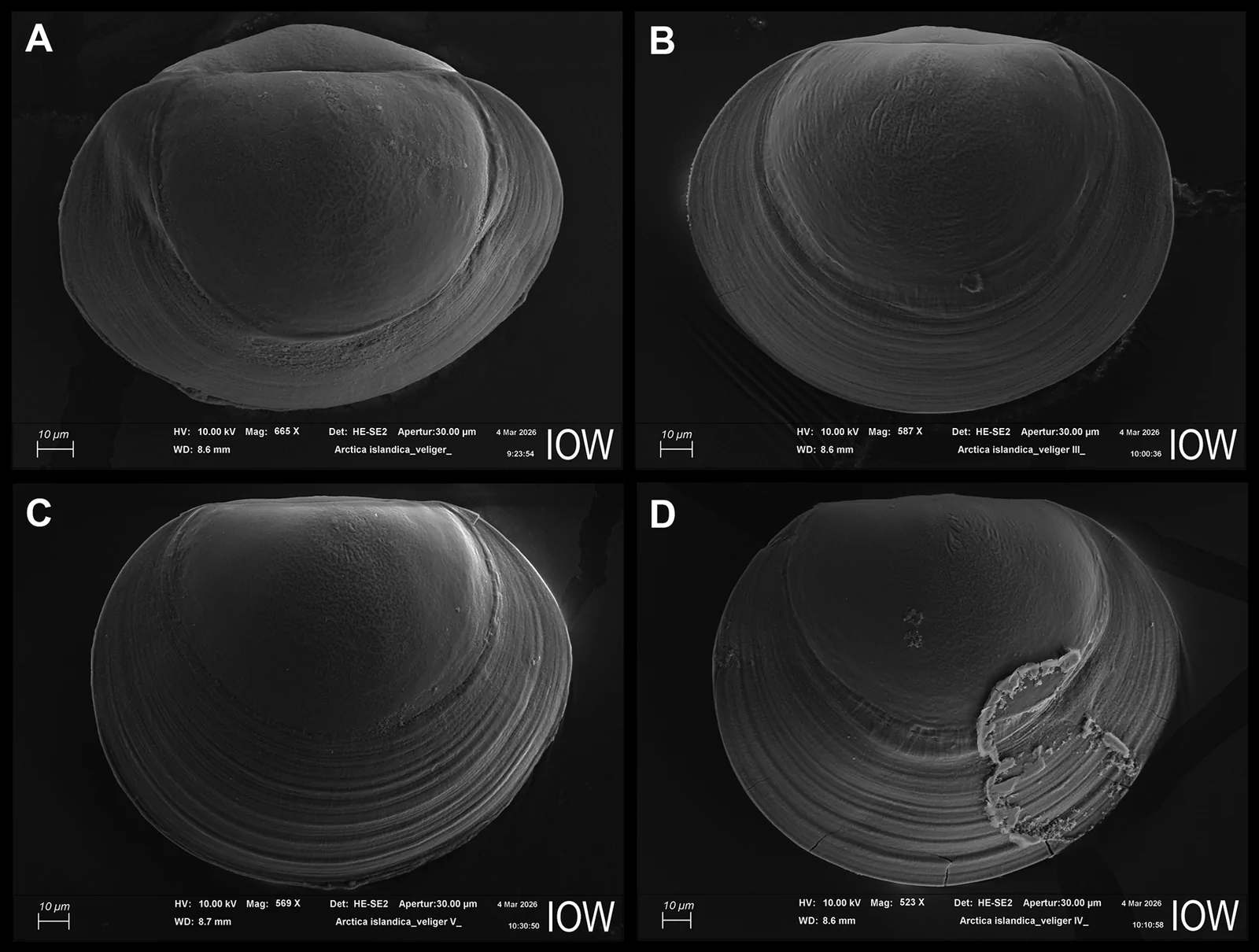
Lateral views of larval shells of *A. islandica* (veliger stage); specimens arranged from (**A**) to (**D**) in ascending order of shell length. **A**. Arko_L101, tilted to show umbonal region; **B–D**. Arko_L103, Arko_L104, and Arko_L105, respectively. Scale bars: 10 µm.

**Internal anatomy. *Mantle***. Lines most of shell cavity, mantle edge visible. ***Velum***. Fan-shaped, nearly filling left half of mantle cavity. ***Adductor muscles***. Anterior adductor muscle visible. ***Foot***. Absent.

#### Pediveliger stage (Figs 2B, 2C, 5, 6; Suppl. material 1: fig. S1)

**Material examined**. • 79 individuals, Baltic Sea, Kiel Bay, 54°23.21520'N, 10°11.86440'E, depth 21.7 m, 22 June 2024, Station KB57; specimens see Suppl. material 1: table SS1.

**Figure 5.**
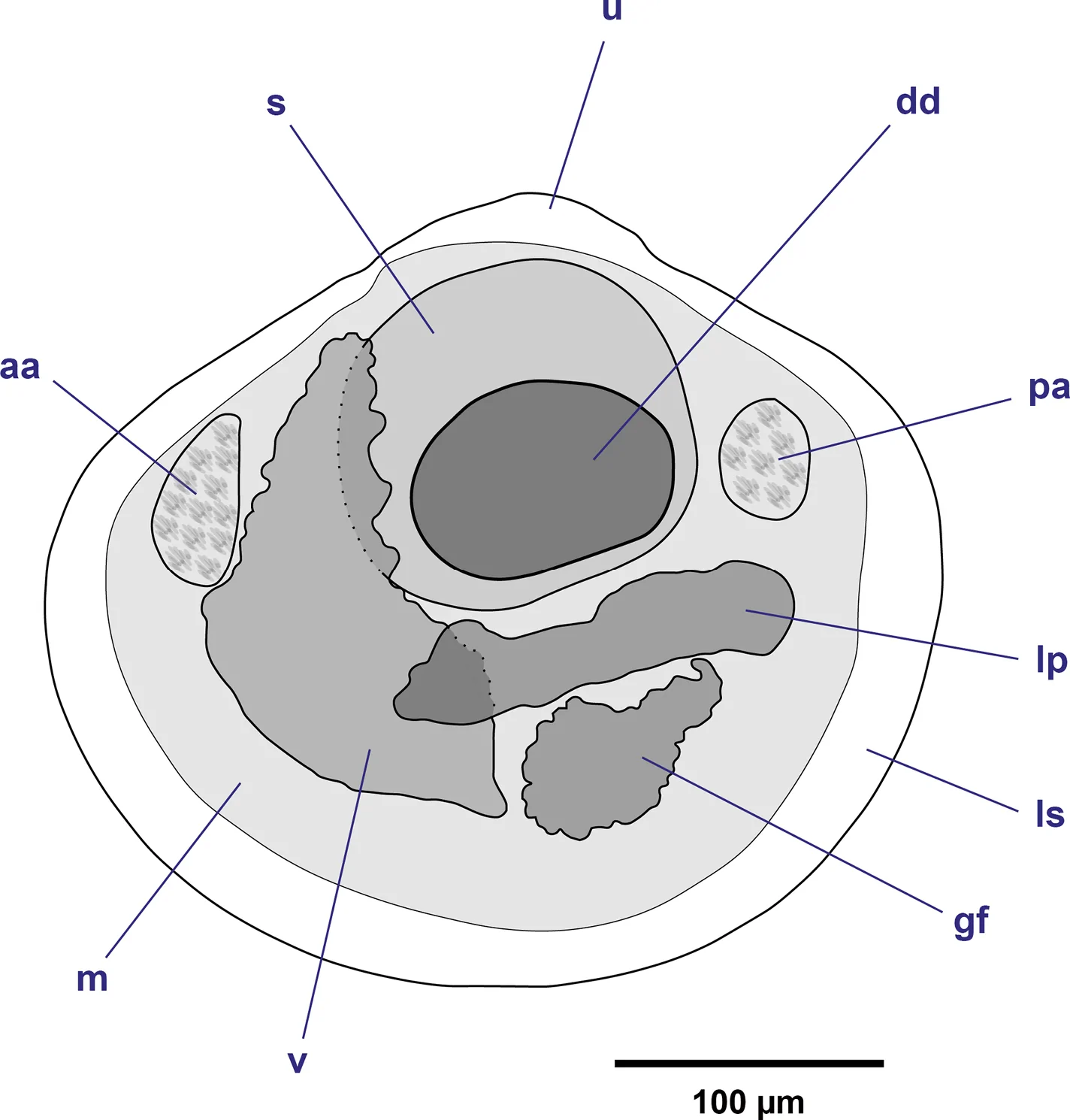
Illustration of pediveliger larva of *A. islandica* (specimen Arko_L089), view from left side of the shell; right: anterior, left: posterior, top: dorsal, bottom: ventral. Abbreviations: s = stomach, u = umbo, dd = digestive diverticulum, pa = posterior adductor, lp = labial palp, ls = larval shell, gf = gill filament, v = velum, m = mantle, aa = anterior adductor.

**Description. *Shell***. Prodissoconch I observed length 88–110 µm, height 64–72 µm (Fig. 5); equivalve, equilateral; ovate outline, with straight, truncated dorsal margin; sculpture of irregular shallow pits and commarginal ridges toward margin; fine radiating lines at margin of prodissoconch II (Fig. 5). Prodissoconch II observed length 217–505 µm (Fig. 2C; mean 338 µm, *n* = 37), equivalve, subequilateral, rounded to elliptical; thin and translucent, slightly yellow; outer ventral area translucent to mantle edge; surface commarginally furrowed; hinge line arched; external ligament and internal teeth absent; for prodissoconch description refer to veliger stage.

**Figure 6.**
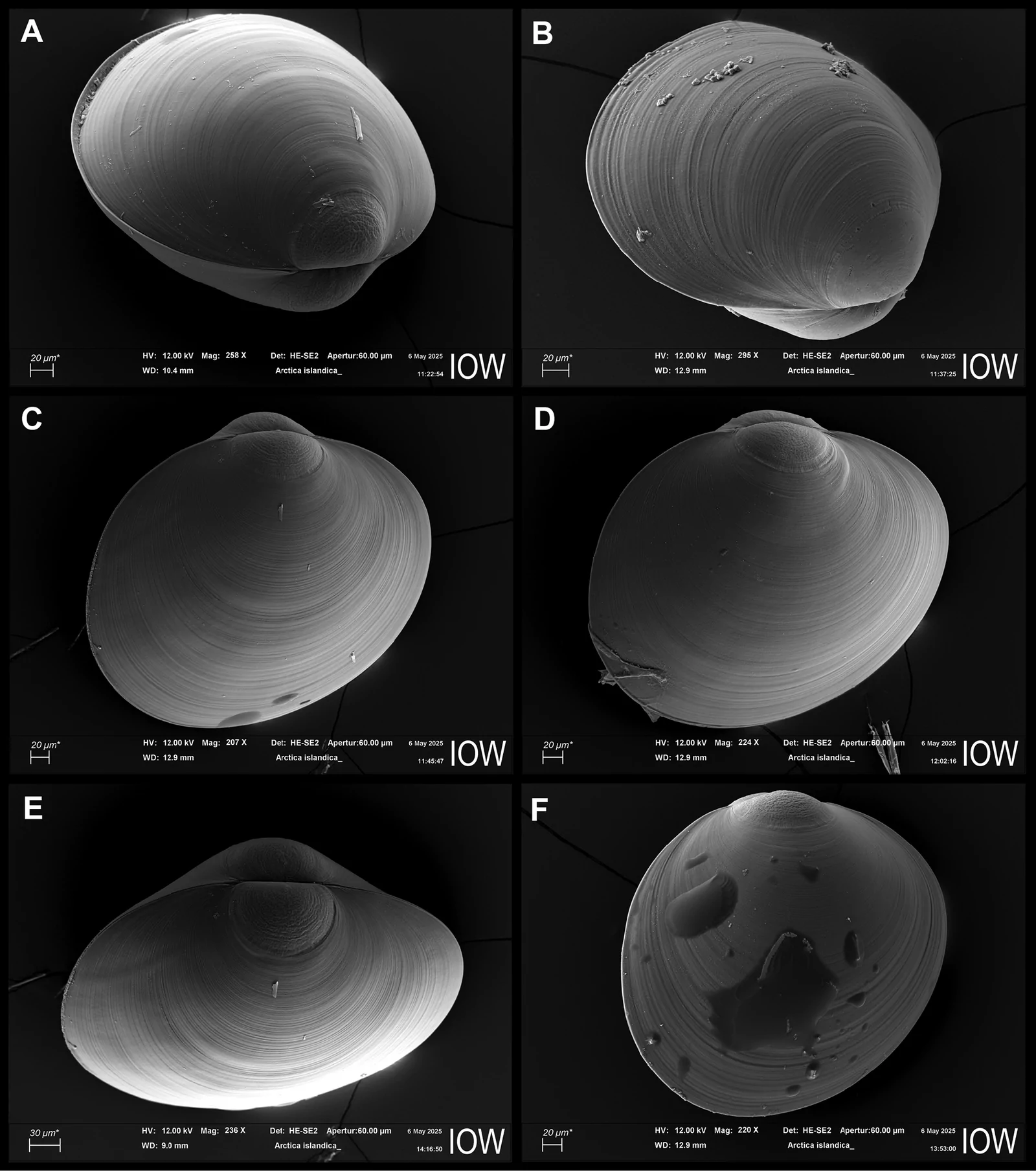
Prodissoconchs of pediveliger larvae of *A. islandica*. **A**. Dorsal view of umbo and hinge (Arko_L096); **B**. Lateral view of larval shell and umbo region (Arko_L097); **C**. View of ventrally positioned shell, strongly arched hinge line (Arko_L098); **D**. View of ventrally positioned shell, slightly curved hinge line (Arko_L099); **E**. View of umbo and hinge (Arko_L098); **F**. Frontal view of right shell (Arko_L100). Scale bars: 20 μm (**A–D, F**); 30 µm (**E**).

**Internal anatomy. *Mantle***. Lines nearly entire shell cavity, mantle edge visible. ***Velum***. Fan-shaped, occupying much of mantle cavity, nearly filling left half. ***Adductor muscles***. Anterior elliptical, posterior more rounded. ***Stomach***. Centrally located, occupying large portion of mantle cavity. ***Digestive diverticulum***. Positioned above stomach, darker and more textured. ***Labial palp***. Elongated; partially overlapping velum and right mantle. ***Gill filament***. Strongly textured, at ventral right edge of mantle. ***Foot***. Not resolved in examined material.

### Description of the larval shell in juvenile animals

Juvenile specimens were examined to identify the retained prodissoconch and provide a reference for shell ontogeny. The individuals are benthic and infaunal.

**Figure 7.**
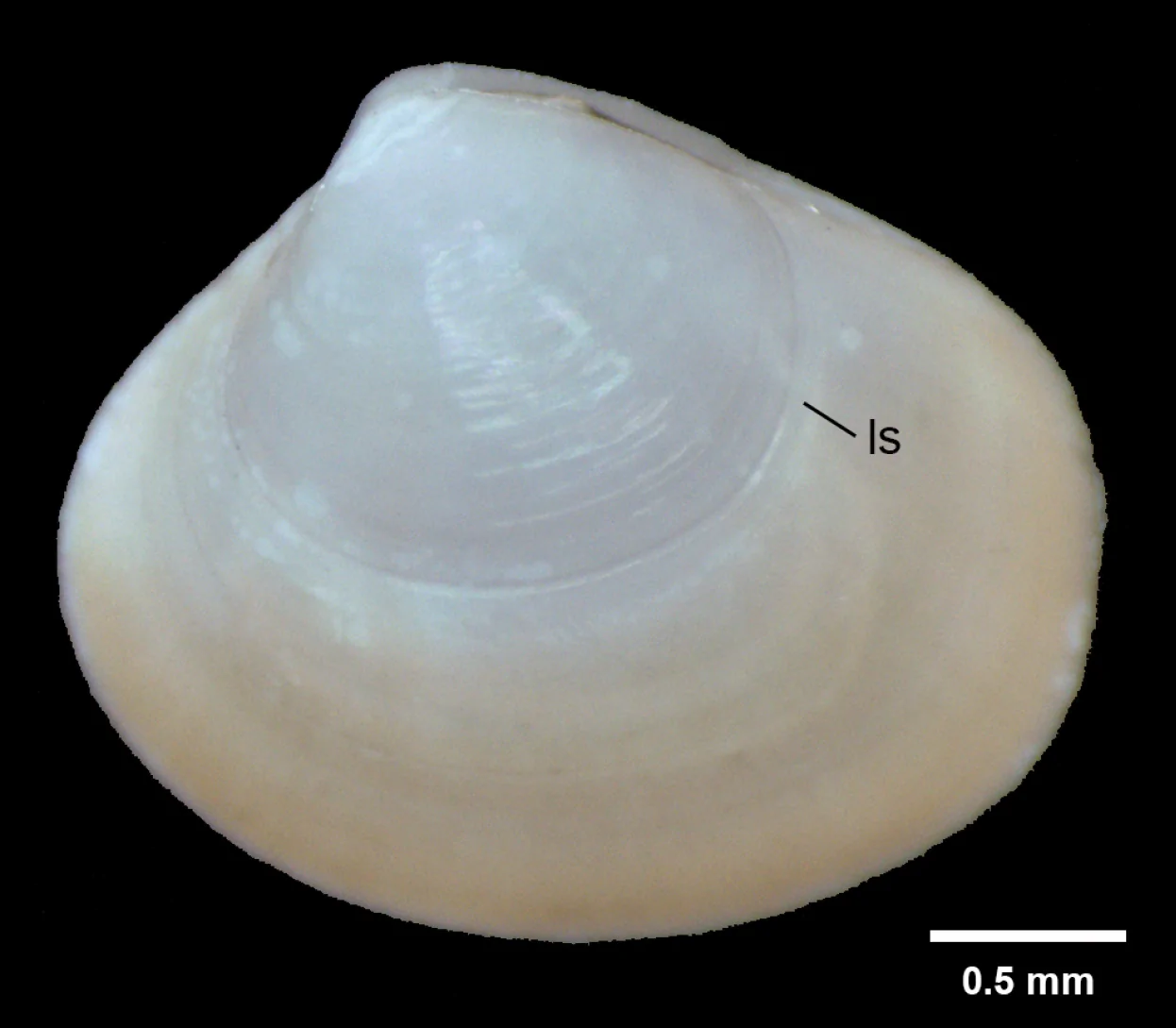
Lateral view of juvenile, benthic individual of *A. islandica* (Arko_451). The margin of the larval shell (ls, prodissoconch II) is marked.

One juvenile specimen of *A. islandica* is shown in Fig. 7. It has a shell length of 3.0 mm. The equivalve, equilateral shell exhibits a clearly visible boundary between two shell portions differing in structure and colouration. The anterior part of the umbo is pearly white and glossy, whereas the shell surface posteriorly and towards the ventral margin is increasingly yellowish. The transition between these two shell regions is marked by a distinct line.

The umbo region is more prominent than in the larval stages described above and leans to the anterior, the posterior dorsal margin is straightened and has an external ligament. The cardinal teeth and a muscular foot are developed. The shell of prodissoconch I remains visible at the umbo, while the subsequent shell growth stage forms the prodissoconch II. Prodissoconch II length is 1452 µm and height is 1293 µm.

Two additional juvenile specimens result in a range for prodissoconch II of 1187–1784 µm in length and 900–1594 µm in height (Suppl. material 1: table S2).

## Discussion

### Genetic validation of larval identity

Larval stages of many marine bivalves, including *A. islandica*, remain poorly described (Hendriks et al. 2005). Thorson (1946) previously illustrated a late pelagic stage presumed to belong to *A. islandica* (then *Cyprina islandica*), but the species’ identity was not verified. Here, genetically validated larval specimens provide the first reliable link between larval morphology and species identity in *A. islandica*, establishing a baseline for future studies of larval development and ecology.

PCR success varied among specimens. Mechanical opening of larval shells prior to DNA extraction has been suggested to facilitate DNA release (Galindo et al. 2014). In the present study, however, successful amplification was obtained from both pierced and intact specimens. This suggests that shell perforation was not the determining factor for PCR success. As the reasons for amplification failure remain unresolved, non-amplification should not be interpreted as evidence that those specimens belonged to a different species.

### Morphological variability and identification challenges

Morphological examinations were conducted alongside the genetic identification of *A. islandica*. Early larval stages are often morphologically similar across bivalve species (Loosanoff et al. 1966; Chanley and Andrews 1971; Garland and Zimmer 2002; Lutz et al. 2018), making species-level identification based solely on morphology challenging. Despite detailed observations, no diagnostic morphological character was identified that would allow unambiguous recognition of *A. islandica* larvae. Apart from shell proportions and the dimensions of prodissoconch I and II, the examined larvae lacked distinctive features such as cardinal teeth, which develop later in ontogeny (Lutz et al. 1982).

The observed larvae closely resembled those of several other bivalve species, including *Mytilus edulis* Linnaeus, 1758; *Geukensia demissa* (Dillwyn, 1817), *Argopecten irradians* (Lamarck, 1819), and *Petricolaria pholadiformis* (Lamarck, 1818), particularly with respect to shell shape (ratio between length and height) (Chanley and Andrews 1971). Furthermore, although shell size has often been used as a taxonomic character, the size range observed in this study differed from previous descriptions of *A. islandica* larvae (Lutz et al. 1982), indicating that size alone is not a reliable identification criterion.

The present study demonstrates that even genetically confirmed *A. islandica* larvae exhibit substantial morphological overlap with descriptions of other bivalve taxa. Consequently, morphology alone cannot be considered sufficient for reliable discrimination among bivalve larval taxa, emphasising the necessity of genetic validation for accurate species assignment.

### Interpretation of developmental stages and comparison with previous descriptions

Larval morphology was compared with Thorson (1946) and, for general bivalve comparison, Bayne (1971; *Mytilus edulis*). Not all internal organs described in previous studies (Thorson 1946; Bayne 1971; Gosling 2007) could be resolved, particularly in veliger larvae, due to tissue density and limitations of microscopy resolution. Fine structures such as the intestine, kidneys, and ganglia were not clearly visible.

The larger specimens (from the June sample) are interpreted as a late developmental stage approaching metamorphosis. Although a foot could not be observed directly, developmental stage assignment in bivalves is generally based on a suite of morphological characteristics rather than on a single diagnostic feature. These larvae exhibit several traits associated with advanced larval development, including large shell size, pronounced umbo development, curved hinge morphology, extensive prodissoconch II growth, differentiation of digestive structures and labial palps, and reduced dominance of the velum within the mantle cavity. Together, these characteristics correspond closely to the pediveliger stage described for *A. islandica* by Lutz et al. (1982). Nevertheless, because a foot could not be visualized directly using the applied methods, the assignment remains an interpretation based on multiple morphological characteristics rather than direct observation of all diagnostic features.

Shell size alone is insufficient for stage assignment and should be interpreted together with additional morphological characteristics. Thorson (1946) described veliger larvae of *A. islandica* up to 325 µm in length, whereas individuals examined in June ranged between 217 and 505 µm. According to Lutz et al. (1982), these sizes are consistent with pediveliger stages, and Lutz et al. reported initiation of metamorphosis at shell lengths exceeding 230 µm. It should be noted that the larvae examined were cultured and were generally smaller (Lutz et al. 1982). The September larvae, with sizes around 162 µm, fall within the veliger range.

A straight hinge line and D-shaped prodissoconch I are characteristic of early veliger larvae (Lutz et al. 1982). Specimens showing these features are interpreted as veligers. In contrast, specimens interpreted as pediveligers exhibited a curved umbo region (Figs 2B, 5, 6) and a more strongly developed hinge area, consistent with a more advanced developmental stage. However, internal morphology could not be documented in comparable detail because of limited visibility, and CLSM imaging did not fully resolve these uncertainties (Suppl. material 1: fig. S1).

The juvenile individuals examined in this study demonstrate the transition from prodissoconch to dissoconch shell formation (Fig. 7), marking metamorphosis (Lutz et al. 1982). Prodissoconch II differs distinctly from both prodissoconch I and the dissoconch in outline, sculpture, and colouration. The dimensions of the preserved prodissoconch II in the juvenile specimens substantially exceeded those observed in the larvae examined here, demonstrating that even the largest larvae collected in this study with a shell length of 505 µm had not yet attained their final larval shell size despite exhibiting several characteristics associated with advanced larval development.

### Seasonal occurrence of *Arctica islandica* larvae

The presence of veliger and pediveliger larvae in different sampling periods indicates that reproduction and larval development of *A. islandica* occur over an extended period. However, because the present study is based on only two positive plankton samples from different locations and years, the data do not allow conclusions regarding the exact timing, frequency, or duration of spawning events.

Temperature is considered one of the major environmental factors influencing reproductive activity and larval development. Loosanoff (1953) reported spawning for *A. islandica* at temperatures around 13.5 °C, while von Oertzen (1972) found mature gametes in Baltic Sea populations over a broad period from March to November. Bottom-water temperatures measured during the present study were 10.4 °C in June and 13.2 °C in September, values that fall within the range associated with reproductive activity. In contrast, the April sample was collected at only 5.6 °C, a temperature considerably lower than those reported during active spawning periods. The absence of larvae in April may therefore reflect delayed reproductive activity and slower larval development under colder conditions.

The occurrence of veliger larvae in September further demonstrates that pelagic development can take place during late summer and early autumn. Such an extended reproductive season has also been suggested for other populations of *A. islandica*, where considerable variability in gonadal development and spawning timing has been observed among individuals (Thorarinsdóttir 2000). Environmental conditions, food availability, and local hydrographic processes may contribute to this variability and influence both spawning success and larval survival.

Nevertheless, larval occurrence in Kiel Bay cannot necessarily be interpreted as direct evidence of local spawning. Water exchange between adjacent areas of the western Baltic Sea may transport larvae over considerable distances, potentially decoupling larval presence from nearby adult populations. Furthermore, recruitment in *A. islandica* is known to be irregular (Powell and Mann 2005), and the limited temporal coverage of the present study precludes assessment of interannual variability. Consequently, the observations presented here should be regarded as evidence of larval occurrence rather than as a basis for detailed conclusions on reproductive phenology.

### Influence of Baltic Sea conditions on larval development

The occurrence of genetically confirmed *A. islandica* larvae in Kiel Bay is consistent with the presence of established adult populations in the Baltic Sea (Brey et al. 1990; Zettler et al. 2001; Schulz et al. 2025). However, little is known about how Baltic Sea conditions influence the early life stages of this species. Compared with populations from fully marine North Atlantic environments, Baltic Sea *A. islandica* exhibit reduced growth rates, thinner shells, and shorter lifespans (Witbaard and Duineveld 1990; Zettler et al. 2001; Sosnowska et al. 2013). Whether similar regional differences occur during larval development remains unresolved.

The larvae documented in this study developed under salinity conditions of 23–24 g/l, substantially lower than those experienced by many North Atlantic populations. Experimental studies have demonstrated that temperature strongly affects developmental rates in *A. islandica* larvae (Landers 1976; Lutz et al. 1981, 1982), whereas the effects of the reduced salinity characteristic of the Baltic Sea remain poorly understood. The discrepancy between larval sizes observed here and those reported from laboratory cultures by Lutz et al. (1982) may indicate environmental influences on growth and development, although methodological differences and uncertainty regarding larval age preclude firm conclusions.

Improved knowledge of larval development and recruitment is particularly relevant because *A. islandica* is considered sensitive to ongoing environmental changes in the Baltic Sea, including warming, deoxygenation, and salinity shifts (Reusch et al. 2018; Schulz et al. 2025). Accurate identification of larval stages, as provided here through genetic validation, represents a prerequisite for future investigations of recruitment dynamics and population persistence under changing environmental conditions.

Several limitations should be considered. Sampling was not designed specifically to investigate reproductive phenology and was limited to three sampling occasions at two locations. Furthermore, larval abundance was not quantified because sampling volume could not be reconstructed. Consequently, ecological interpretations should be treated cautiously.

Future studies combining genetic identification with histological or advanced imaging approaches may help resolve remaining uncertainties regarding late larval development and metamorphosis.

## Conclusion

This study provides the first genetically confirmed description of *A. islandica* larvae. Morphologically distinct larval forms were confirmed belonging to the same species, demonstrating the limitations of morphology-based identification and the importance of genetic validation. The larger larvae are interpreted as late developmental stages consistent with pediveligers, although a foot could not be observed directly. These findings provide a baseline for future studies on the early life history, larval ecology, and conservation of this exceptionally long-lived bivalve.

## Additional information

### Artificial Intelligence (AI) use

The authors accept full responsibility for the content of the manuscript, including the disclosure of any use of AI.

No AI tools were used in the preparation of this manuscript.

### Author ORCIDs

C.A. Chaibi https://orcid.org/0009-0009-9019-0461

K. Kniesz https://orcid.org/0000-0003-0697-5418

M.L. Zettler https://orcid.org/0000-0002-5437-5495

### Data availability

The generated sequences have been uploaded to GenBank (https://www.ncbi.nlm.nih.gov/genbank/) and BOLD (https://doi.org/10.5883/DS-ARKO01). Accession numbers are provided in Material and methods and Suppl. material 1: table SS1. All additional metadata (photographs, station information, raw sequence data) are as well provided in BOLD.

## Supplementary materials

Supplementary material 1 Supplementary tables and figure Data type docx Explanation note Contains data tables with the information on the studied material, including the GenBank accession numbers, measurements, PCR and sequencing success rates. In addition, a confocal laser scanning image is presented, which shows the internal structure of the pediveliger larvae. https://binary.pensoft.net/file/1762721This dataset is made available under the Open Database License (http://opendatacommons.org/licenses/odbl/1.0/). The Open Database License (ODbL) is a license agreement intended to allow users to freely share, modify, and use this Dataset while maintaining this same freedom for others, provided that the original source and author(s) are credited. Chiraz A. Chaibi, Katharina Kniesz, Michael L. Zettler
