# Supplementary material for: Why genetic validation is crucial for identifying larval stages of Arctica islandica (Venerida, Arcticidae)
Source: Zookeys. 2026 Sep 2;1291:55–73. doi: 10.3897/zookeys.1291.195659 (PMC13559521; doi:10.3897/zookeys.1291.195659)
Supplement: Supplementary file 1 [file zookeys-1291-055_article-195659__-s001.docx]

ZOOKEYS

**Genetically validated description of veliger and pediveliger larvae of *Arctica islandica* in the Baltic Sea**

Chiraz A. Chaibi^1^, Katharina Kniesz^2^, Michael L. Zettler^2^

^1^ Universität Rostock, Rostock, Germany.

^2^ Leibniz-Institut für Ostseeforschung Warnemünde, Rostock, Germany.

# Corresponding author: Katharina Kniesz (katharina.kniesz@iow.de)

# **Supplementary Material**

**Supplementary Table S1:** Overview of all examined larvae of *A. islandica* from Kiel Bay (Baltic Sea), including the three juvenile specimens. Provided are the individual sample code, station number, measurements of the shell (length and height), method of morphological study, S = Scanning Electron Microscopy (SEM), C = Confocal Laser Scanning Microscopy (CLSM), L = light microscopy, D =drawing, M = molecular analysis. GenBank accession numbers and Barcode of Life Datasystems (BOLD) number are provided.

| Station number | Coordinate [Lat., Long.] | Sampled month | Development stage | Sample code | Length [µm] | Height [µm] | Applied method (S/C/L/D/M) | GenBank accession number | BOLD number |
| --- | --- | --- | --- | --- | --- | --- | --- | --- | --- |
| KB102 | 54° 32.52' N;  10° 40.47' E | September 2024 | veliger | Arko_L086 | - | - | M | - | - |
|  |  |  |  | Arko_L087 | - | - | M | - | - |
|  |  |  |  | Arko_L088 | 170 | 139 | L/D/M | PZ721711 | ARKOB017-25 |
|  |  |  |  | Arko_L093 | 161 | 119 | S | - | - |
|  |  |  |  | Arko_L094 | 175 | 112 | S | - | - |
|  |  |  |  | Arko_L095 | 147 | 134 | S | - | - |
|  |  |  |  | Arko_L101  x | 143 | 110 | S | - | - |
|  |  |  |  | Arko_L102  3 | 152 | 127 | S | - | - |
|  |  |  |  | Arko_L103 | 156 | 124 | S | - | - |
|  |  |  |  | Arko_L104  3 | 160 | 131 | S | - | - |
|  |  |  |  | Arko_L105 | 168 | 141 | S | - | - |
|  |  |  |  | Arko_L106 | 185 | 149 | S | - | - |
| KB57 | 54° 23.22' N;  10° 11.86' E | June 2024 | pediveliger | Arko_L001 | 505 | 457 | L/M | - | - |
|  |  |  |  | Arko_L002 | - | - | M | - | - |
|  |  |  |  | Arko_L003 | - | - | M | PZ721701 | ARKOB001-25 |
|  |  |  |  | Arko_L004 | - | - | M | - | - |
|  |  |  |  | Arko_L005 | - | - | M | - | - |
|  |  |  |  | Arko_L006 | - | - | M | - | - |
|  |  |  |  | Arko_L007 | - | - | M | PZ721710 | ARKOB002-25 |
|  |  |  |  | Arko_L008 | - | - | M | - | - |
|  |  |  |  | Arko_L009 | - | - | M | PZ721699 | ARKOB003-25 |
|  |  |  |  | Arko_L010 | - | - | M | PZ721698 | ARKOB004-25 |
|  |  |  |  | Arko_L011 | - | - | M | - | - |
|  |  |  |  | Arko_L012 | - | - | M | - | - |
|  |  |  |  | Arko_L013 | - | - | M | - | - |
|  |  |  |  | Arko_L014 | - | - | M | - | - |
|  |  |  |  | Arko_L015 | - | - | M | - | - |
|  |  |  |  | Arko_L016 | - | - | M | PZ721706 | ARKOB005-25 |
|  |  |  |  | Arko_L017 | - | - | M | - | - |
|  |  |  |  | Arko_L018 | - | - | M | - | - |
|  |  |  |  | Arko_L019 | - | - | M | PZ721697 | ARKOB006-25 |
|  |  |  |  | Arko_L020 | - | - | M | - | - |
|  |  |  |  | Arko_L021 | - | - | M | PZ721704 | ARKOB007-25 |
|  |  |  |  | Arko_L022 | - | - | M | - | - |
|  |  |  |  | Arko_L023 | - | - | M | - | - |
|  |  |  |  | Arko_L024 | - | - | M | - | - |
|  |  |  |  | Arko_L025 | - | - | M | - | - |
|  |  |  |  | Arko_L026 | - | - | M | PZ721695 | ARKOB008-25 |
|  |  |  |  | Arko_L027 | - | - | M | - | - |
|  |  |  |  | Arko_L028 | - | - | M | - | - |
|  |  |  |  | Arko_L029 | - | - | M | - | - |
|  |  |  |  | Arko_L030 | - | - | M | - | - |
|  |  |  |  | Arko_L031 | - | - | M | - | - |
|  |  |  |  | Arko_L032 | - | - | M | - | - |
|  |  |  |  | Arko_L033 | - | - | M | - | - |
|  |  |  |  | Arko_L034 | - | - | M | - | - |
|  |  |  |  | Arko_L035 | - | - | M | - | - |
|  |  |  |  | Arko_L036 | - | - | M | - | - |
|  |  |  |  | Arko_L037 | - | - | M | PZ721700 | ARKOB009-25 |
|  |  |  |  | Arko_L038 | - | - | M | - | - |
|  |  |  |  | Arko_L039 | - | - | M | - | - |
|  |  |  |  | Arko_L040 | - | - | M | - | - |
|  |  |  |  | Arko_L041 | - | - | M | - | - |
|  |  |  |  | Arko_L042 | - | - | M | - | - |
|  |  |  |  | Arko_L043 | - | - | M | - | - |
|  |  |  |  | Arko_L044 | - | - | M | - | - |
|  |  |  |  | Arko_L045 | - | - | M | - | - |
|  |  |  |  | Arko_L046 | - | - | M | - | - |
|  |  |  |  | Arko_L047 | - | - | M | - | - |
|  |  |  |  | Arko_L048 | - | - | M | - | - |
|  |  |  |  | Arko_L049 | - | - | M | - | - |
|  |  |  |  | Arko_L050 | - | - | M | - | - |
|  |  |  |  | Arko_L051 | - | - | M | - | - |
|  |  |  |  | Arko_L052 | 252 | 220 | L/M | - | - |
|  |  |  |  | Arko_L053 | 375 | 344 | L/M | - | - |
|  |  |  |  | Arko_L054 | 401 | 361 | L/M | - | - |
|  |  |  |  | Arko_L055 | - | - | M | - | - |
|  |  |  |  | Arko_L056 | 416 | 391 | L/M | - | - |
|  |  |  |  | Arko_L057 | 301 | 273 | L/M | - | - |
|  |  |  |  | Arko_L058 | 217 | 191 | L/M | - | - |
|  |  |  |  | Arko_L059 | 400 | 380 | L/M | PZ721702 | ARKOB010-25 |
|  |  |  |  | Arko_L060 | 431 | 393 | L/M | - | - |
|  |  |  |  | Arko_L061 | 296 | 259 | L/M | - | - |
|  |  |  |  | Arko_L062 | 276 | 238 | L/M | - | - |
|  |  |  |  | Arko_L063 | - | - | M | - | - |
|  |  |  |  | Arko_L064 | 295 | 273 | L/M | PZ721708 | ARKOB011-25 |
|  |  |  |  | Arko_L065 | 369 | 351 | L/M | - | - |
|  |  |  |  | Arko_L066 | 312 | 285 | L/M | - | - |
|  |  |  |  | Arko_L067 | 314 | 270 | L/M | PZ721705 | ARKOB012-25 |
|  |  |  |  | Arko_L068 | 352 | 328 | L/M | - | - |
|  |  |  |  | Arko_L069 | 327 | 304 | L/M | - | - |
|  |  |  |  | Arko_L070 | 291 | 259 | L/M | - | - |
|  |  |  |  | Arko_L071 | 299 | 270 | L/M | PZ721709 | ARKOB013-25 |
|  |  |  |  | Arko_L072 | 272 | 245 | L/M | - | - |
|  |  |  |  | Arko_L073 | 316 | 284 | L/M | PZ721696 | ARKOB014-25 |
|  |  |  |  | Arko_L074 | 273 | 267 | L/M | - | - |
|  |  |  |  | Arko_L075 | 336 | 327 | L/M | - | - |
|  |  |  |  | Arko_L076 | 385 | 353 | L/M | PZ721703 | ARKOB015-25 |
|  |  |  |  | Arko_L077 | 308 | 279 | L/M | PZ721707 | ARKOB016-25 |
|  |  |  |  | Arko_L078 | 380 | 320 | L/M | - | - |
|  |  |  |  | Arko_L079 | 296 | 283 | L/M | - | - |
|  |  |  |  | Arko_L080 | 264 | 240 | L/M | - | - |
|  |  |  |  | Arko_L081 | 253 | 239 | L/M | - | - |
|  |  |  |  | Arko_L082 | - | - | M | - | - |
|  |  |  |  | Arko_L083 | - | - | M | - | - |
|  |  |  |  | Arko_L084 | 423 | 391 | L | - | - |
|  |  |  |  | Arko_L085 | 378 | 312 | L | - | - |
|  |  |  |  | Arko_L089 | 342 | 302 | L/D | - | - |
|  |  |  |  | Arko_L090 | - | - | C | - | - |
|  |  |  |  | Arko_L096 | 369 | 247 | S | - | - |
|  |  |  |  | Arko_L097 | 294 | 266 | S | - | - |
|  |  |  |  | Arko_L098 | 377 | 317 | S | - | - |
|  |  |  |  | Arko_L099 | 401 | 304 | S | - | - |
|  |  |  |  | Arko_L100 | 414 | 341 | S | - | - |
| EMB320 | 56° 51.97' N;  11° 26.01' E | July 2023 | juvenile | Arko_450 | 2226 | 1959 | L | - | - |
|  |  |  |  | Arko_451 | 2843 | 2345 | L | - | - |
|  |  |  |  | Arko_452 | 3161 | 3610 | L | - | - |

**Supplementary Table S2:** Measurements of prodissoconch I and II of larval and juvenile specimens and the hinge length. The prodissoconch II corresponds to the total shell length in both larval stages.

| Sample Code | Prodissoconch I | | Prodissoconch II | | Hinge Lenth [µm] |
| --- | --- | --- | --- | --- | --- |
|  | Length [µm] | Height [µm] | Length [µm] | Height [µm] |  |
| Veliger stage | | | | | |
| Arko_L093 | - | - | 155 | 119 | 66 |
| Arko_L094 | - | - | 163 | 112 | 76 |
| Arko_L095 | 111 | - | 147 | 134 | 65 |
| Arko_L101 | 102 | 80 | 143 | 110 | 60 |
| Arko_L102 | 106 | 80 | 152 | 127 | 75 |
| Arko_L103 | 123 | 91 | 156 | 124 | 76 |
| Arko_L104 | 115 | 78 | 160 | 131 | 72 |
| Arko_L105 | 115 | 86 | 168 | 141 | 80 |
| Arko_L106 | 113 | 84 | 185 | 149 | 76 |
| Pediveliger stage | | | | | |
| Arko_L096 | 88 | 70 | 369 | 247 | 66 |
| Arko_L097 | 94 | 68 | 294 | 266 | - |
| Arko_L098 | 100 | 68 | 377 | 317 | 67 |
| Arko_L099 | 105 | 64 | 401 | 304 | 73 |
| Arko_L100 | 110 | 72 | 414 | 341 | - |
| Juvenile stage | | | | | |
| Arko_450 | - | - | 1784 | 1594 | - |
| Arko_451 | - | - | 1452 | 1293 | - |
| Arko_452 | - | - | 1187 | 900 | - |

**Supplementary Figure S1:** Internal anatomy of pediveliger larva of *A. islandica* (specimen Arko_L090), view from left and right side; photographs by confocal laser imaging (CLSM). **(dd)** digestive diverticulum, **(ls)** larval shell, **(u)** umbo, **(v)** velum.

**Supplementary Table S3:** PCR and sequencing success rates for the studied larvae. Included is the larval stage, treatment before DNA extraction (pierced/unpierced), samples, total number N, number of available bands in agarose gel, resulting sequences and high-quality sequences after processing (minimum length 490 bp, minimum HQ 90%),

| Extraction/ PCR | Larval stage | Treatment | Samples | N | Bands | Sequences | High-quality sequences |
| --- | --- | --- | --- | --- | --- | --- | --- |
| 1 | pediveliger | pierced | Arko_L001-Arko_L051 | 51 | 24 | 15 | 9 |
| 2 | pediveliger | unpierced | Arko_L052-Arko_L085 | 31 | 7 | 7 | 7 |
| 3 | veliger | unpierced | Arko_L086-Arko_L089 | 3 | 1 | 1 | 1 |
| Total |  |  |  | 85 | 32 | 23 | 17 |
